# Supplementary material for: Modeling glioblastoma heterogeneity as a dynamic network of cell states
Source: Mol Syst Biol. 2021 Sep 16;17(9):e10105. doi: 10.15252/msb.202010105 (PMC8444284; doi:10.15252/msb.202010105)
Supplement: Supplementary file 6 — Source Data for Figure 5 [file MSB-17-e10105-s004.zip › Figure5A_sourcedata/GSEA_3071/hallmarks_stateII.GseaPreranked.1623742536692/gsea_report_for_na_pos_1623742536692.html]

Report for na\_pos 1623742536692 [GSEA]

| GS  follow link to MSigDB | GS DETAILS | SIZE | ES | NES | NOM p-val | FDR q-val | FWER p-val | RANK AT MAX | LEADING EDGE || 1 | HALLMARK\_E2F\_TARGETS | Details ... | 157 | 0.71 | 4.57 | 0.000 | 0.000 | 0.000 | 750 | tags=76%, list=18%, signal=90% |
| 2 | HALLMARK\_G2M\_CHECKPOINT | Details ... | 133 | 0.63 | 3.99 | 0.000 | 0.000 | 0.000 | 656 | tags=63%, list=16%, signal=73% |
| 3 | HALLMARK\_MYC\_TARGETS\_V1 | Details ... | 107 | 0.49 | 3.07 | 0.000 | 0.000 | 0.000 | 1507 | tags=72%, list=37%, signal=111% |
| 4 | HALLMARK\_MITOTIC\_SPINDLE | Details ... | 101 | 0.47 | 2.84 | 0.000 | 0.000 | 0.000 | 744 | tags=47%, list=18%, signal=55% |
| 5 | HALLMARK\_MYC\_TARGETS\_V2 | Details ... | 40 | 0.52 | 2.55 | 0.000 | 0.000 | 0.001 | 1610 | tags=83%, list=39%, signal=135% |
| 6 | HALLMARK\_DNA\_REPAIR | Details ... | 67 | 0.44 | 2.39 | 0.000 | 0.000 | 0.001 | 536 | tags=37%, list=13%, signal=42% |
| 7 | HALLMARK\_SPERMATOGENESIS | Details ... | 28 | 0.44 | 1.92 | 0.002 | 0.007 | 0.039 | 451 | tags=39%, list=11%, signal=44% |
| 8 | HALLMARK\_EPITHELIAL\_MESENCHYMAL\_TRANSITION | Details ... | 95 | 0.31 | 1.85 | 0.000 | 0.010 | 0.064 | 881 | tags=49%, list=21%, signal=62% |
| 9 | HALLMARK\_ALLOGRAFT\_REJECTION | Details ... | 35 | 0.40 | 1.83 | 0.002 | 0.011 | 0.079 | 948 | tags=46%, list=23%, signal=59% |
| 10 | HALLMARK\_MTORC1\_SIGNALING | Details ... | 99 | 0.30 | 1.80 | 0.000 | 0.012 | 0.094 | 1507 | tags=60%, list=37%, signal=92% |
| 11 | HALLMARK\_IL2\_STAT5\_SIGNALING | Details ... | 67 | 0.29 | 1.62 | 0.012 | 0.045 | 0.329 | 992 | tags=45%, list=24%, signal=58% |
| 12 | HALLMARK\_ESTROGEN\_RESPONSE\_LATE | Details ... | 67 | 0.29 | 1.58 | 0.018 | 0.053 | 0.392 | 844 | tags=43%, list=21%, signal=54% |
| 13 | HALLMARK\_APICAL\_JUNCTION | Details ... | 66 | 0.29 | 1.58 | 0.016 | 0.050 | 0.401 | 756 | tags=33%, list=18%, signal=40% |
| 14 | HALLMARK\_UNFOLDED\_PROTEIN\_RESPONSE | Details ... | 41 | 0.32 | 1.56 | 0.050 | 0.053 | 0.440 | 805 | tags=37%, list=20%, signal=45% |
| 15 | HALLMARK\_INFLAMMATORY\_RESPONSE | Details ... | 50 | 0.27 | 1.40 | 0.056 | 0.121 | 0.761 | 448 | tags=26%, list=11%, signal=29% |
| 16 | HALLMARK\_UV\_RESPONSE\_DN | Details ... | 68 | 0.23 | 1.24 | 0.167 | 0.273 | 0.972 | 874 | tags=35%, list=21%, signal=44% |
| 17 | HALLMARK\_ESTROGEN\_RESPONSE\_EARLY | Details ... | 65 | 0.23 | 1.23 | 0.171 | 0.265 | 0.976 | 813 | tags=35%, list=20%, signal=43% |
| 18 | HALLMARK\_PEROXISOME | Details ... | 31 | 0.26 | 1.18 | 0.257 | 0.323 | 0.994 | 498 | tags=23%, list=12%, signal=26% |
| 19 | HALLMARK\_TNFA\_SIGNALING\_VIA\_NFKB | Details ... | 86 | 0.20 | 1.18 | 0.229 | 0.309 | 0.994 | 1197 | tags=43%, list=29%, signal=59% |
| 20 | HALLMARK\_GLYCOLYSIS | Details ... | 73 | 0.21 | 1.15 | 0.247 | 0.330 | 0.997 | 916 | tags=33%, list=22%, signal=42% |
| 21 | HALLMARK\_ANDROGEN\_RESPONSE |  | 44 | 0.21 | 1.04 | 0.412 | 0.489 | 1.000 | 608 | tags=25%, list=15%, signal=29% |
| 22 | HALLMARK\_UV\_RESPONSE\_UP |  | 53 | 0.18 | 0.95 | 0.556 | 0.620 | 1.000 | 1241 | tags=47%, list=30%, signal=67% |
| 23 | HALLMARK\_WNT\_BETA\_CATENIN\_SIGNALING |  | 15 | 0.26 | 0.90 | 0.582 | 0.667 | 1.000 | 790 | tags=33%, list=19%, signal=41% |
| 24 | HALLMARK\_PI3K\_AKT\_MTOR\_SIGNALING |  | 36 | 0.18 | 0.85 | 0.689 | 0.729 | 1.000 | 198 | tags=11%, list=5%, signal=12% |
| 25 | HALLMARK\_INTERFERON\_GAMMA\_RESPONSE |  | 46 | 0.17 | 0.83 | 0.709 | 0.729 | 1.000 | 434 | tags=15%, list=11%, signal=17% |
Table: Gene sets enriched in phenotype **na **[plain text format]****
